# Bath-Ambience—A Mechatronic System for Assisting the Caregivers of Bedridden People

**DOI:** 10.3390/s17051156

**Published:** 2017-05-18

**Authors:** Karolina Bezerra, José Machado, Vítor Carvalho, Marcelo Castro, Pedro Costa, Demétrio Matos, Filomena Soares

**Affiliations:** 1 MEtRICs Research Center, School of Engineering, University of Minho, 4800-058 Guimarães, Portugal; karolceli@dem.uminho.pt (K.B.); dmatos@ipca.pt (D.M.); 2 Mechanical Engineering Department, School of Engineering, University of Minho, 4800-058 Guimarães, Portugal; marcelovieiracastro@gmail.com (M.C.); a65391@alunos.uminho.pt (P.C.); 3 R&D ALGORITMI Centre, School of Engineering, University of Minho, 4800-058 Guimarães, Portugal; vcarvalho@ipca.pt (V.C.); fsoares@dei.uminho.pt (F.S.); 4 IPCA, Polytechnic Institute of Cávado and Ave, Vila Frescainha S. Martinho, 4750-810 Barcelos, Portugal

**Keywords:** wellbeing, mechatronic design, smart environment, caregiver, bathing system

## Abstract

The health of older people is receiving special attention and dedication nowadays, with the aim of increasing their general wellbeing and quality of life. Studies into different aspects of the care of the elderly have found that emphasis should be given to solving problems related to bathing in different situations and environments. In particular, it is important to develop new assistive technologies to streamline and ease the burden of a caregiver’s daily tasks. Generally-speaking, in the case of bedridden patients, bathing is typically carried out manually by a caregiver, using towels, sponges, and a water basin. Nevertheless, this apparently simple task needs some precautions in order to avoid the risk of microbial infections, falls and other injuries. With that in mind, this paper presents the design of a portable washing system, called Bath-Ambience, which enables bedridden patients to be bathed efficiently without having to be moved from their position. This portable system can be installed in different situations, both in a domestic setting, and in specialized institutions, and allows the caregiver to perform the bathing tasks without compromising health and safety, thereby making it possible to offer a comfortable and hygienic procedure to patients, improving their quality of life. This paper presents the design of the portable Bath-Ambience washing system, which provides efficient assistance for bathing bedridden patients without moving them to another place. This system is mainly dedicated for integration a smart home application in to allow bathing everywhere.

## 1. Introduction

One of the greatest current concerns of developed countries is associated with the increase of the number of elderly people. According to the Japanese Health, Labour and Welfare Report [1], it is stated that one of five persons is currently a senior citizen, sixty-five years of age or older, and the estimation is that by 2020 one in three persons will be a senior citizen. It is also estimated that by 2055 the proportion of senior citizens will exceed forty percent of the whole population [1,2].

The elderly population requires extreme caution in relation to their activity either in home bath installations or those in an institution, from the perspective of providing an improved quality of life for both caregivers and elderly people. To reduce eventual or serious illnesses or even death different technological assistance or monitoring of persons in the home using sensors deployed around houses, creating smart houses, and, in this case, smart baths [3,4,5,6].

Considering this, reference [2] states that healthcare includes all the goods and services designed to promote health. It can be considered a complex system that integrates and combines people, processes, and products [3]. Of course, healthcare as a whole will benefit from any major upgrade provided by means of newly developed devices.

The bathing of bedridden patients is among the various daily care tasks that is also one of the most difficult. It requires physical effort from the caregiver in order to perform the sequence of movements demanded in the activity. It is also necessary to analyze the degree of dependence of the patient’s condition. For example, if the patient is severely limited in his/her capacity to move, more effort and assistance are required, thereby, justifying the development of a device that can assist with the task of moving and placing the patient in the bath [6,7,8].

Therefore, the need to have a healthy ageing society requires major help from technology [3,4,5,6,7,8,9,10,11,12,13,14,15,16,17,18,19,20,21,22,23,24,25,26,27]. Considering technologies developed to assist the activities to care for bedridden people, there have been several developments in the bathing area, such as improving the the environment for giving the bath, smart bathrooms [5,6,7,8], bed technology [14,15], washing systems [16,17,18,19], including the use of body sensors and control systems [20,21,22,23,24]. Usually, in a domestic environment, there is only one caregiver taking care of the bedridden person. Thus, dedicated devices should provide the full sup-port to undertake the required healthcare tasks efficiently, thus increasing the autonomy, independency, and the quality of life of potential elderly people.

In order to assist the domestic caregivers in giving baths to bedridden people, smart baths—portable washing systems have been designed; these work without having to move the patient to the nearest bathroom or water source. The system can be an integral part of the smart house, as part of the environment of care function to promote a proof-of-concept for the elderly bath task improvement. During the design process some main ideas have been taken into account and, always, a critical behavior of authors has been adopted [28].

The structure of the proposed system consists of two water tanks standing one on top of the other, both filled through the same hole on the top of the tank. One of the tanks contains cold water, while the other houses a secure heating system controlled by appropriate sensors. A controller is installed in order to mix the water from both tanks and to adjust the temperature to the liking of the patient, bearing in mind the temperature intervals that health-professionals advise. Another part of the system is the shower, which was chosen to fulfill the requirements of providing adjustable pressure and temperature settings.

This paper is structured in five sections. The first one is the Introduction. The second section, State of the Art, consists of a contextualization of the project, motivation, and description of technologies and systems suitable for use for bedridden people. In the third section, Framework, and in the fourth section, Results, the system components and their operation are described, along with a short explanation on how to use the interface and a systematic explanation of the use of the designed product. In section five, Conclusions, the main conclusions of the study as well as some future steps of the project are presented.

## 2. State of the Art

It is important to mention that this project represents a technical innovation inside the Ambient Assisted Living (AAL) paradigm, offering and increasing opportunities for an independent and self-determined way of life for older people living at home or in care facilities [9,10,11].

With an aging population, there is a deterioration of the physical capabilities of both bedridden persons and caregivers, and this aspect is of utmost importance to determine how independent the patient is. Furthermore, the psychological state is also responsible for variations in the quality of life. The healthcare of people with total motion disability makes the caregiver work exhausting, resulting many times in the appearance of health problems in the caregiver due to repetitive stress. In healthcare for the elderly, some aspects such as physical fragility and skin care need to be considered among others, because of the deterioration of the condition of the skin with aging.

The requirements to use an AAL-developed project provided a strong conceptual basis. The functionalities of this mechatronic system to assist the bathing will support impaired functions of potential users. This project has a relevant background in the various assistive areas: bathing, comfort and security. These help develop a suitable environment to care for older persons. The knowledge was acquired during studies in this area and bearing in mind the increasing demands regarding assistive technologies to help in the bathing situation.

It is important to agree on the importance of the security parameters to be included in any product to be used by care-givers in care environments. Knowledge about sensor technology, communication systems, and information technologies has created ample opportunities to develop novel equipment facilitating care for the elderly. Smart homes equipped with wireless sensor networks will benefit both health care providers and their patients. In this project, sensors are applied to ensure the security of the use of the system and improve aspects like quality of life for elderly people such as privacy, independence, dignity and convenience, which are supported and enhanced by the ability to provide services in the patient’s own home [12].

Using Information and Communication Technology (ICT)−based products and services for the propose to increase the benefits for older adults, developers and service providers must not only focus on the functionality itself, but also, even especially, on the user interface. Part of the purpose was to provide a generic framework for the design of flexible user interfaces, such as: AAL, GPII/URC and universal ones [13].

This represents a proof-of-concept of equipment to help bathe bedridden people. In this case, commercial sensors were implemented to prototype this concept, and maybe in the future will be necessary to develop off-the-shelf sensors to have specifics functions. For example, technological solutions have been created to help in daily care situations. Medical Care Terminal (MCT) + BED, is a system that collects physiological data of the patient and provides a remote medical interface, allowing medical instructions to be transmitted to the patient or caregiver. Environment variables can also be monitored by the system [14]. The environment for giving an automated bath was developed to meet the different demands of patients; therefore, it provides an environment that supports the patient in standing in a vertical position, which makes it possible to run a pre-programmed course of a sequence of tasks: lathering, rinsing and drying, in order to minimize the effort of caregivers [5,6,7,8,9,10,11,12,13,14,15,16,17,18,19,20,21,22,23].

In a specific hospital bath project [6], designed to provide the bathing of an invalid or of a patient in the hospital, allowing maximum mobility and minimal patient discomfort, a vertical support was included that enables users to choose different height settings.

Furthermore, to assist the caregivers to reduce the burden of nursing care, several implementations were investigated in order to identify limitations and possible areas for improvement. In particular, the work performed by [8] describes three different solutions used currently. The first is an electric wheelchair that can be modified in order to work as a portable bed by reclining the seat. The second is a new type of lift for carrying the patient from the wheelchair to the bath, or to a normal bed. The third component is a new type of bathtub made from vinyl cloth and supported by an aluminum frame that can be moved easily.

Another project supported daily showering, via a manual or electric device fitted with a disposable sleeve coupled in a sliding support, which can move in the vertical and horizontal directions, in order to facilitate lathering. It is important to highlight the operation of the electrical system, which uses an internal bar attached to the support, a toothed belt and an engine that is driven by an external button that allows the holder to raise or decrease the limit end-of-stroke, while also including intensity control [16].

Similarly, a patented system to enable better conditions related to sitting in the bed, using an inflatable mattress with a brush that performs movements under the patient’s back was developed. It has water drainage channels, which allow washing the surface. The system controls the inflation of the air mattress and the drying process [17].

In a recent project [18], a bathing system for assisted and non-independent bedridden patients it was developed with the use of an inflatable PVC air tub, which is universal and suitable for any bed. This system allows bathing by means of a common water dispenser, and characterized by a main inflatable and ergonomic central body, a headrest coplanar with the main body, which supports the bather, and a periphery tubular air wall with three functions. The main functions are safety of the bather against accidental falls; water splash containment during bathing; and canalization of wastewater towards the drain hole. One important aspect of the construction of the air tub is that it allows the patient to remain in a raised position during the washing, thus ensuring better hygiene as the body is not immersed in water.

Another implementation considers a mobile service on a bed equipped with a waterproof material with raised edges forming a bath with a tank used to hold water for washing the patient and holding a therapeutic liquid. The service tank can be used to pump water with pressure, or to regulate the flow rate of supplied water and the water pressure in the shower at the end of the supply line; a control valve is preferably in the feed line of the tank provided. This equipment can be connected to electrical units either using either a battery, or to AC power, with a rechargeable battery [19].

**Figure 1:**
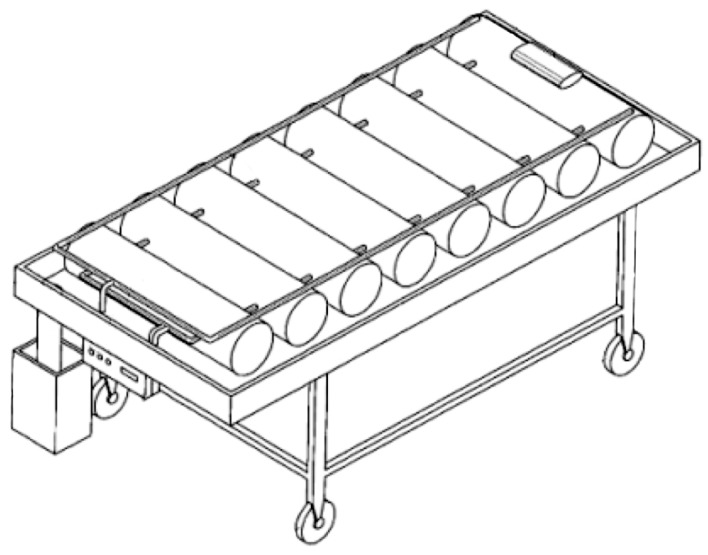
Air cushion bed with water—draining device (adapted from [13]).

In 2002, a system which consisted in a bed with air cushion, with water working as drainage device was patented (Figure 1, [20]). The system integrates an air pump, an air duct, and a controller. The structure contains a multiple air mattress with a series of holes in different directions; the controller activates the pump for conveying air, controls the filling and emptying of the mattress. The base plate of the water tank is inclined and it has a water outlet in the lower part. Therefore, the effluent contained in the water reservoir can be channeled through the inclined base and discharged through the water outlet, allowing functions of body wash and cleaning of the excrements in bed. The air ducts are responsible for ensuring both cold and hot air, allowing the user to keep warm during winter and keep cool during summer; it can be used by disabled or patients in an immobile state [20].

**Figure 2:**
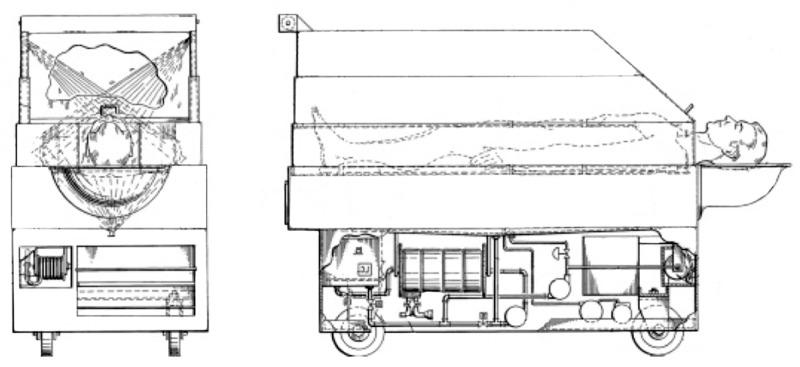
Bathing device (adapted from [14]).

Another 1975 patent [21] described a bathing system specifically for immobilized patients (Figure 2). This device can automatically wash and dry the patient without the physical constant presence and attention of the nurses. Patients can be easily positioned in the shower thus optimizing healthcare processes.

Another washing system was developed to help caregivers shower bedridden people [22]. This concept of a mechatronic system is composed by two parts, the SmartBath (SB) and Bath Control System (SCB). It is a unique system with improvements in terms of comfort, accessibility, safety and ease of use directed to the elderly and their caregivers. The system may improve the users’ quality of life, allowing accessibility, privacy, and safety.

A different project was designed to minimize the difficulty of the caregiver in moving the bedridden patient from the bed to an intelligent bathroom, including bath monitoring based on technologies such as bioimpedance analysis, water level and temperature control and detection, and the activity of the person in the bathroom [23].

Other example considers an exoskeletal robot for humans with joints designed to match those of the wearer—“Robot Suit HAL” [24]. The study focused on reducing the trunk inclination angle, which is related to the physical load around the lumbar spine. A mechanism that locks the power unit of a Hybrid Assistive Limb (HAL) joint is added to the caregiver’s upper limb used to support the weight of the care-receiver to assist the motor function of the wearer’s upper limbs [24].

Furthermore, modern technology has allowed the development of miniaturized wireless mobile health monitoring systems, capable of continuous monitoring while being power efficient, such as the Bathtub controller [25]. These systems are able of monitoring the patients in rehabilitation, the elderly in assisted living, and patients with chronic diseases and allow frequent measurements of physiological parameters on a daily basis in everyday life [25].

After the analysis of the above systems, it can be stated that there is a gap in mobile bath care devices. Mobility enables a higher quality of care for bedridden in the daily activities carried out by caregivers, especially in the bath activity. Therefore, in this paper it is proposed the development of a portable device allowing bathing in any environment with the best conditions, in order to improve the quality of life of caregivers.

## 3. Framework

This section presents the Portable Washing System (PWS). The concept of the product intends to promote mobility in order to give showers to bedridden people, allowing the wellbeing and hygiene of users. In this section it is explained how the PWS works, how and why it was designed, as well the methodology for establishing functional classifications and design decisions on a value analysis basis. The methodology considers the basic principles of the product development [29,30]. The process shows that technologies, specifications and functions have the power to enable a project in many ways. A step-by-step product development methodology was applied and is presented in the following subsections.

The PWS was conceived to reduce the effort of caregivers, while meeting all the requirements of a conventional bath. This equipment is composed by a main device plugged into the electric current supply where the water is heated, contained and pumped to the shower and an air bed with an outlet for the water flow placed under the patient, on the bed.

As main advantage, besides the cost, it is important to know that the temperature of the water is an important parameter to control in order to prevent injuries and complications during the bath. The selected water temperature controller is programmed to allow working temperatures between 38 °C and 42 °C, allowing the caregiver to select the temperature between these values and adjust it during the bath.

Moreover, the modular design of the system allows easy maintenance. Its modular parts facilitate the access to the various components and the parts are all removable, which facilitates the repair of each part individually, without damaging the product. The device can be used in any home and/or hospital environment. The mechanism enables quick operation due to the system developed to supply and heat water. The reservoir tank requires the use of a common water supply method. This procedure is performed in about 8 min considering the average flow rate of 5 L/min and carried out in approximately 22 min using a power of 3 KW resistance. In this line of reasoning, this means a caregiver can have the product ready to give the bath in 30 min, a relatively good time, taking into account the current total time of about 1 h.

The first step is to connect a hose to a water supply, such as a tap; this will fill the two recipients without the need to connect both to the water supply. Secondly, the equipment is turned on and starts heating the water to the maximum recommended temperature. After this procedure, it is necessary to hold the shower, turn it on, and regulate the temperature using the display on the side of the tank. Then, the pressure and flow of the water in the shower need to be adjusted accordingly.

In case of failure of one of the components, after diagnosing the failure and identifying the component, it is not necessary to dismantle all the system to repair that part. The advantage of the modular design is that the caregiver does not need to take the equipment to a technician in order to clean the water filter, for example, as the system was designed to allow an easy access to every component. Comparing the proposed system to a traditional bath, the advantages of the PWS presented in this paper are:

- “Bath in Bed”—for patients with total dependence on a caregiver;
- Easier for the caregiver to change environments (house or hospital);
- Quick water supply and water heating;
- Ease of use;
- No risk of falling, no risk of damaging the skin, safe water temperature;
- Low cost equipment.

In terms of ease of use, the system was designed with a tactile interface for easy understanding and operation of the system, in order to allow the user to choose the parameters of the prototype, such as filling water tank, temperature control, water pressure, water supply, and warming time.

### 3.1. Concept Requirements

Taking into account that the device focuses on assisting caregivers in their activities of giving baths to bedridden patients, a system composed by a structure allowing heating water and respective transportation between spaces was developed, composed by two stainless steel tanks with an insulating layer of polyurethane.

One of the tanks has a capacity of 30 L and it is destined to heat water up to 43 °C. It is composed by two resistances (each one of 1500 W), but it can also work with just one of them, allowing to the caregiver to choose the duration for warming the water. The other tank has a capacity of 15 L and it is used to store cold water.

By using two water pumps, the system pumps the water to the piping system through a thermostatic valve with a controller that allows the caregiver to choose a bath temperature between 38 °C and 42 °C (as previously mentioned). This temperature limitation prevents unnecessary injuries. The product specifications are summarized in Table 1.

### 3.2. Functional Decomposition

The process of creating a design architecture for the Bath-Ambiance device followed a process of decomposition, in which a top-level concept of the system’s required functions is broken down into functions and after into sub-functions. According to Ullman [28] two perspectives can be adopted: the decomposition of each function that is partitioned into sub-functions and the requirements allocated to that function that need to be decomposed with it.

**Figure 3:**
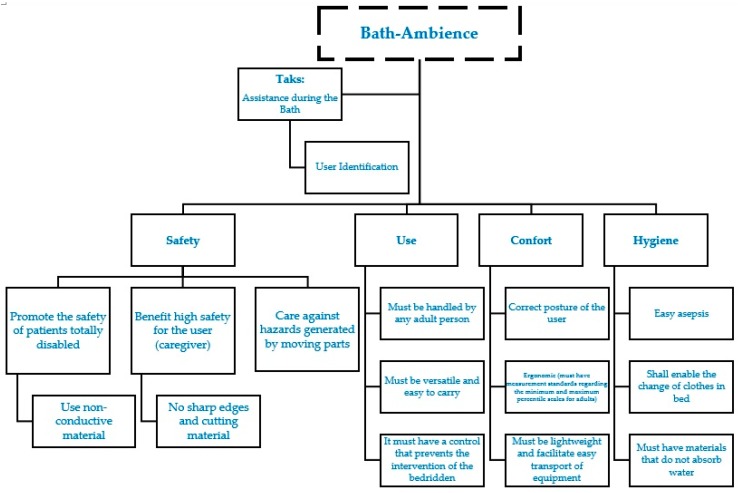
Functional decomposition [29].

From these perspectives we obtain a decomposition of the functions according to the areas of relevance for the development of the prototype. This proposal has the main task of providing assistance during the bath, and in the line of concept development were defined a series of objectives focused on the users, who are the caregivers. In this case the areas defined for the in depth study of the functions are: Safety, Use, Comfort and Hygiene (Figure 3).

Each function requires deep understanding in functional and ergonomic terms. For example the Comfort function that requires that when the equipment it will be possible to adapt it to the weight of a user and ensure the correct posture of the user.

In the Safety area the functions consist of promoting the safety of totally disabled patients and a sub-function consists of using non-conductive materials. The function “benefit high safety for the user” which sub-function is “no sharp edges and cutting material” is devoted to the safety of the caregiver. The last one is “care against hazards generated by moving parts” that covers possible worries of both the bedridden patient and the caregiver. The Use area has just functions such as “Must be handled by any adult person”, “Must be versatile and easy to carry”, and “It must have a control that prevents the intervention of the bedridden”. These functions address the main worries about correct use of the equipment, taking into account the specific conditions of caring for a bedridden person by a caregiver. In the Comfort area, the functions are “correct posture of the user”, “ergonomic” (must have measurement standards regarding the minimum and maximum percentile scales for adults) and it must be lightweight to facilitate easy transport of the equipment. Finally, the last area, Hygiene, the function “easy asepsis” defines how to enable changing clothes in bed and the use of materials that do not absorb water. All functions and sub-functions are implemented in the equipment. Therefore, it can be concluded that the development of this equipment requires the accomplishment of the functions, presented in Table 2, described in this step based on the definition of the guidelines for its detailed design and consequent construction.

### 3.3. Morphological Chart

The technique, here presented, uses all the identified functions to foster ideas. It is a very powerful method that can be formally used, as presented here, or informally as part of everyday thinking. There are two steps to this technique. The goal of the first step is to find as many concepts as possible that can provide each function identified in the decomposition. The second step is to combine these individual concepts into overall concepts that meet all the functional requirements. Just the second one was used in this work.

The design engineer’s knowledge and creativity are crucial here, as the ideas generated are the basis for the remainder of the design evolution. This technique is often called the “morphological method”, and the resulting table (see Table 2) is called a “morphology”, which means “a study of form or structure” [28,29].

The purpose of this study is defined as providing the best understanding of how a device can be constructed. The parts that integrate the product design must follow some functions and requirements to provide the most added value to the proposed Bath—Ambience device. The complete conceptual design considers the selection of one concept for each function and combines all those selected into a single solution. Following the development of the PWS, it is possible to show which criteria were used to choose each one for each function of this concept.

The wheels need to have load capacity up to 2.5 times the safety coefficient for an estimated bearing weight of 80 kg. It must be made of a sturdy material and allow the equipment to be strong, safe and multidirectional and backward fixed.

Regarding the tanks and their water capacity it is estimated that a water outlet operated at 5 L per minute with an inconstant flow rate, to achieve a bath duration of 8 min needs a tank of approximately 30 L capacity for 40 min.

To promote the mobility of the device, it must support the user, and it is intended that the support be ergonomic to allow it to interact with the wheels and it must be able to support all the integrated parts up to 80 kg in weight.

The mixing valve integrated inside of system was chosen as an In-Line configuration, Digital LED Thermostatic Mixing Valve System. This solution comes with a LED Monitor connected to the valve that allows the caregiver to regulate the temperature during the bath without the need of someone more qualified to access the valve. Comparing to the Bath Mounted Thermostatic Mixing Valve this is a better solution since the valve position before the system outlet allows a new level of temperature control to ensure the patient safety.

The water filter choice is one of these solutions that should be made according to what aspect of the water needs to be treated. The chosen solution was to treat the water at the entrance of the system to filter out chlorine. The filtration system solution to use is integrated in the Whole-house chlorine filters category.

Finally, the shower head function was chosen by having a Dynamic Shower Outlet control, possible flow control, easy adjustment and a comfortable handle.

**Figure 4:**
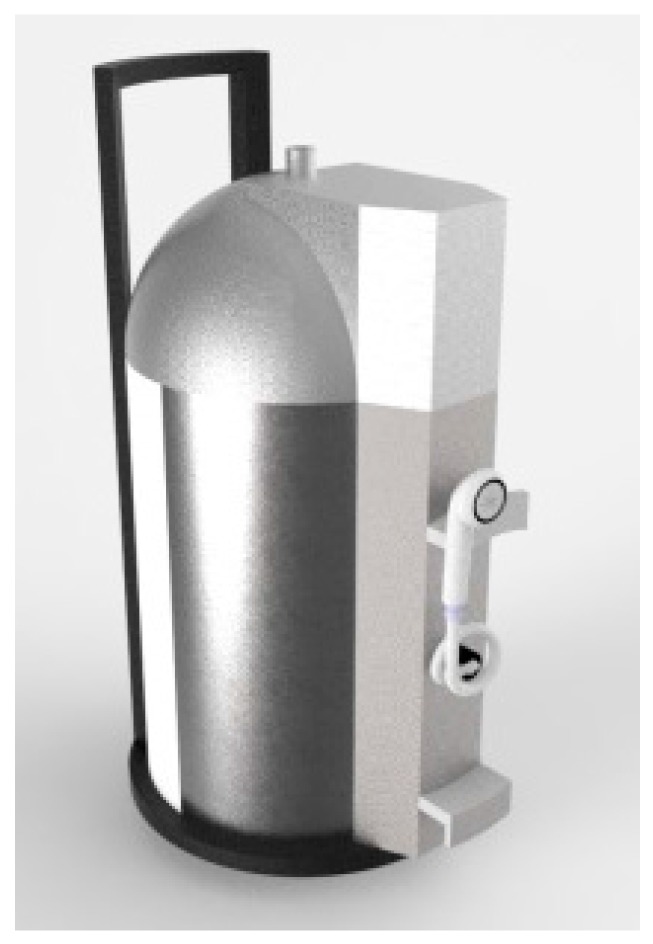
Conceptual model of the 3D Portable Washing System.

Figure 4 presents the virtual modeling of the PWS developed by the conceptual design. The final proof-of-concept is described in the next subsections.

### 3.4. Developed Prototype

**Figure 5:**
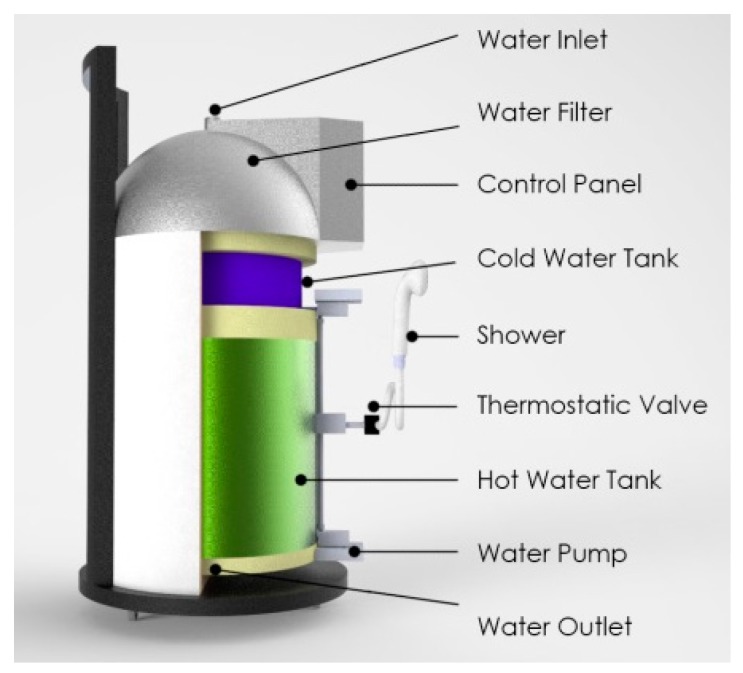
Final prototype (water inlet, water filter, control panel, cold water tank, shower, thermostatic valve, hot water take, water pump, water outlet).

A 3D model of the prototype was developed taking into account the dimensions necessary to incorporate all the electronics and meet the full set of requirements. The prototype was idealized for easy transportation (avoiding problems such as issues crossing doors in private houses). The dimensions include a base with a diameter of 544 mm and a height of 900 mm. The equipment was designed to be easy to use by both healthcare professionals and common citizens in the comfort of his/her home. The system was divided into subsystems, in order to make it easier to dismantle the system, as well as facilitating the design and development of the system. Figure 5 presents all parts of the system.

### 3.5. Water Tanks

The tanks are one of the most important components of the system. It is the place to which the hoses connect and where the electric resistance and most of the other components are installed. As such, special attention was given to their design. Firstly, it was decided that both tanks needed to be transported everywhere, in order to give the caregiver all the independence and mobility that a system like this could provide. Secondly, they were designed to be hygienic and durable, with no rust or other particles, from the beginning to the end of the expected life of the component. Finally, the tanks had to keep the water inside at a given temperature, in order to ensure a safe and comfortable bath. When it come to the materials used, the main aim was to make the system affordable, without compromising its specifications. Thus, a stainless steel case was chosen, while polyurethane foam ensured the insulation. This way, the system was both visually appealing and functional, without affecting its cost or quality of the medical care.

### 3.6. Insulation

In the tank design it was intended to ensure a very good thermal insulation, so it was decided that the water temperature should not decrease by more than 1 °C over the course of three hours.

A structure with a stainless steel outer shell, and with polyurethane as the main insulator, was considered. Table 3 lists the water and environmental temperature, as well as the tank’s capacity and dimensions. The thermal properties of water, air, and raw materials analyzed and used are shown in Table 4.

To improve and certify this mechatronic system a series of mathematical studies were performed so it was possible to test various thicknesses. Each thickness was calculated, as well as the possible new values of *Q* (heat transfer). The maximum power lost is defined by the thermal energy that is needed to drop the water temperature by 1 °C over the course of one hour. This could be calculated based on the following Equation (1):

(1) $$Q=\frac{p\times V\times Cp\times \Delta T}{\Delta t}$$

where *p* is the density (kg/m³), *Cp* is the heat capacity at constant pressure (J/(kg·K)), Δ*T* is the variation in temperature (K), *V* is the velocity (m/s), and Δ*t* is the time interval (s).

The heat transfer value returned was 17.42 W and it was used in Equation (2) as *Qmax*. The stainless steel thickness is standardized; for a heater tank wall it must be from 1.4 to 2 mm, but in this particular case the maximum thickness of 2 mm was chosen. Then, the temperature of the interior of the tank surface was determined using Equations (2) and (3):

(2) $$Qmax=\frac{Tin-Tis}{Rcvi}$$

(3) Tis=Tin−Teff/(hw×Ais)

*Qmax* (W) represents the maximum heat transfer, *Tin* (K) is the temperature of the exterior of the tank surface, *Tis* (K) is the temperature of the interior of the tank surface, and *R* (K·m/W) is the specific thermal resistance.

After this step, the effective power loss was calculated for each thickness of the insulation layer. The procedure required calculating the outside radius of the insulation layer for each insulation thickness. This leads to Equation (4) for the area of the outer surface:

(4) $$Aos=\pi \times {\left(r_{Tss}+T_{PU}\right)}^{2}\times 2+2\times \pi \times \left(r_{Tss}+T_{PU}\right)\times \left(z+2\times T_{PU}\right)$$

Here *Aos* represents the outside surface area, *ss* represents stainless steel and *pu* corresponds to the polyurethane. Next, the temperature of the outer face of the insulator was calculated, employing Equations (5) and (6):

(5) $$Qmax=\frac{Tos-Tenv}{\frac{1}{hair\times Aos}}$$

(6) $$Tos=Tenv+\frac{Qmax}{h_{air}\times Aos}$$

where *Tos* is the temperature of the outside surface area; *Tenv* is the temperature of the environment; and *Aos* is the outside surface area, and hair is the enthalpy.

Having determined both temperatures, it was considered the heat conduction of the stainless steel and the polyurethane walls. Thus, for each thickness interaction, the effective losses were calculated, considering distinct flows on the top and sides of the tank, as in Equations (7)–(9):

(7) Qef=Qcil+Qplain

(8) $$Qef=\frac{Tis-Tes}{R_{ss.cil}+R_{PU.cil}}+\frac{Tis-Tes}{R_{ss.plain}+R_{PU.plain}}$$

(9) $$Qef=\frac{Tis-Tes}{\frac{\ln\left(\frac{r_{T.ss}}{r_{i.ss}}\right)}{2\times \pi \times Tss\times kss}+\frac{\ln\left(\frac{r_{T.PU}}{r_{T.ss}}\right)}{2\times \pi \times Tss\times k_{PU}}}+\frac{Tis-Tes}{\frac{Tss}{k_{ss}\times Ass}+\frac{T_{PU}}{k_{PU}\times A_{PU}}}$$

Above, *Qef* is the final heat flow of the tank; *T* is the temperature; *k* is the thermal conductivity; and A is the area. In conclusion, in order to achieve the desired insulation and considering a stainless steel thickness of 2 mm, a layer of polyurethane with a thickness of 30 mm must be used [28,29].

### 3.7. Resistences

In order to ensure safe and comfortable bath for the user, temperature becomes a key factor. According to Thermostatic Mixing Valve Manufacturers Association, and in order to avoid burns on the patient’s skin, the temperature cannot exceed 42 °C, and it cannot drop below 38 °C, because it can lead to irregular heartbeat and breathing problems. To heat the water to the desired temperature, was considered the use of a resistance and a thermostat. Water heating is accomplished by using an electrical resistance. This kind of system is also used in conventional water heaters and it is very reliable and safe, as it does not release gases related to combustion systems, for example. Its design is simple, and it is easy to introduce and easy to control.

After choosing the system, it was needed to select a resistance. It was assumed that preparing a bedridden person for a bath usually requires from 20 to 25 min. As such, it was considered that this time interval would be sufficient to heat the water. The expression that allows calculating the necessary power to heat the fluid is presented in Equation (10):

(10) $$Q=\frac{m\times Cp\times \Delta T}{t}$$

*Q* required to cool or heat within a specific time interval is calculated in relation to *m* (mass of water), *Cp* (specific heat of water), Δ*T* (temperature variation), and *t* (time required to heat).

The *Cp* of water in these conditions is 4180 J/(kg·°C). Its total mass is 30 kg and the temperature difference is, in extreme conditions, of 32 °C (water filled at 10 °C being heated up to 42 °C). Two electric resistances were used, considering their size, cost and electrical consumption from the grid. Based on these factors, two resistances of 1500 watts each were chosen. The calculated heating interval was approximately 22 min, which matches the above value [31,32,33,34,35].

#### 3.7.1. Water Pump

**Figure 6:**
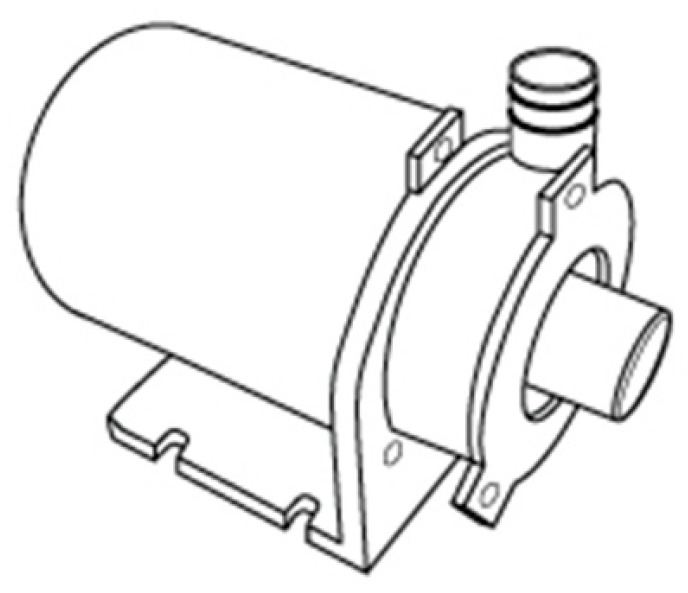
Water pump model (adapted from [36]).

The system is composed by two water pumps, each one placed in the water outlet of each water tank. The water pump selected is presented in Figure 6.

This water pump has a pumping capacity of 6.8 L/min with a pressure of 4.33 PSI (0.298542 bar) which is enough taking into account that the desired final water flow of the system is 5 L/min.

#### 3.7.2. Water Filter

**Figure 7:**
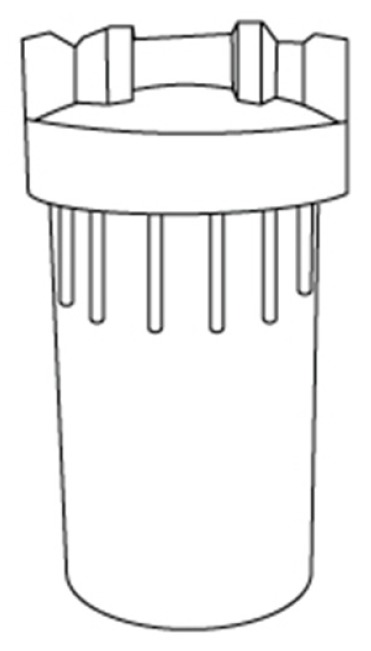
Water filter (adapted from [37]).

The water from the mains may contain impurities, so the use of a water filter is important in order to remove possible impurities in the pipes, as well as high levels of chlorine, which could be uncomfortable to the bedridden. For these reasons, a water filter was selected (Figure 7), which is set up at the entry of water in the tanks. It is recommended to clean the filter after each bath, to ensure a constant quality of water.

#### 3.7.3. Water Temperature Control

The water temperature control is based on mixing two different water flows with known temperatures: the cold water flow and the hot water flow [38]. To determine the relation between the flows, there are some principles that need to be assumed in order to simplify the calculations and respected in order to assure a correct final equation of flows. In this part is determined the equation of water flows, considering:

- Cold Water Temperature: Tc
- Hot Water Temperature: TH
- Mixed Water Temperature: TO.

The following principles were assumed, in order to simplify the calculations:

Flow in steady state:

(11) $$\Delta m_{VC}=0;\;\Delta E_{VC}=0$$

Negligible Kinetic Energy and Potential Energy:

(12) $$E_{C}=E_{P}=0$$

- No interaction of work: The conservation of mass it is an important tool used in the analysis of physical systems. To determine the mixing flow, the mass balance and the energy balance provide important equivalences in the calculation of the final flow equation:

Mass Balance:

(13) $$\Delta m_{sistema}=m_{adm}=m_{sai}$$

(14) $$m_{adm}=m_{sai}\to m_{1}+m_{2}=m_{3}$$

Energy Balance:

(15) $$E_{system}=E_{adm}-E_{sai}$$

$E_{adm}-E_{sai}$: Rate of energy transferred by heat, work, and mass transfer.

$E_{system}$: Rate of change of kinetic energy, potential energy, ...

(16) $$E_{adm}=E_{sai}$$

(17) $$m_{1}\times h_{1}+m_{2}\times h_{2}=m_{3}\times h_{3}$$

Assuming the aforementioned principles (*Q* = 0; *W* = 0; *E_C* = *E_P* = 0):

(18) $$m_{1}\times h_{1}+m_{2}\times h_{2}=(m_{1}+m_{2})\times h_{3}$$

(19) $$\frac{m_{1}h_{1}}{m_{2}}+\frac{m_{2}h_{2}}{m_{2}}=\frac{(m_{1}+m_{2})\times h_{3}}{m_{2}}$$

With the next relation (*γ*), to simplify the Equation (19):

(20) $$\gamma =\frac{m_{1}}{m_{2}};\;\gamma \times h_{1}+h_{2}=\left(\gamma +1\right)\times h_{3}$$

Using the next expression, it is possible to know the relation between the hot water and cold water flows (*γ*):

(21) $$\gamma =\frac{h_{3}-h_{2}}{h_{1}-h_{3}}$$

#### 3.7.4. Thermostatic Valve

**Figure 8:**
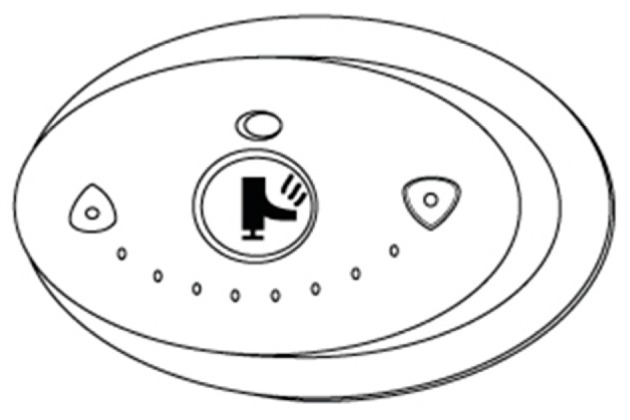
Thermostatic valve (adapted from [31]).

**Figure 9:**
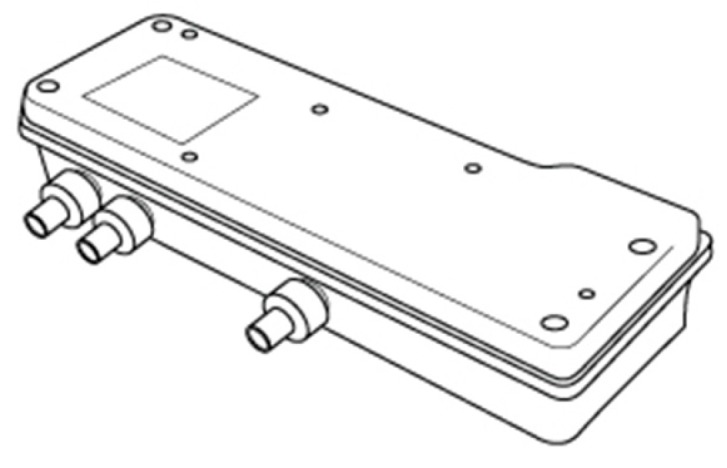
Temperature controller (adapted from [31]).

The thermostatic valve selected was an Armstrong Sense DMV2-Individual Shower (Figure 8). This valve allows setting the minimum and/or maximum temperature values. Thus, it is accompanied by a control panel which will be programmed for temperatures between 38 °C and 42 °C (±1 °C) [8]. It is, also, used a temperature controller as indicated in Figure 9.

**Figure 10:**
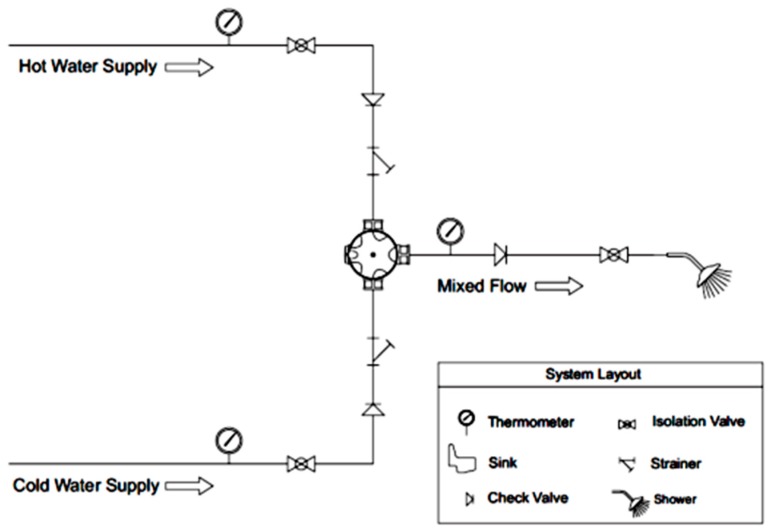
Thermostatic valve scheme [31].

Figure 10 presents the scheme of the valves and the control checkpoints of temperature to assure that the mixed flow had the correct temperature, in order to prevent temperatures outside the range of 38–42 °C.

#### 3.7.5. Sensors

**Figure 11:**
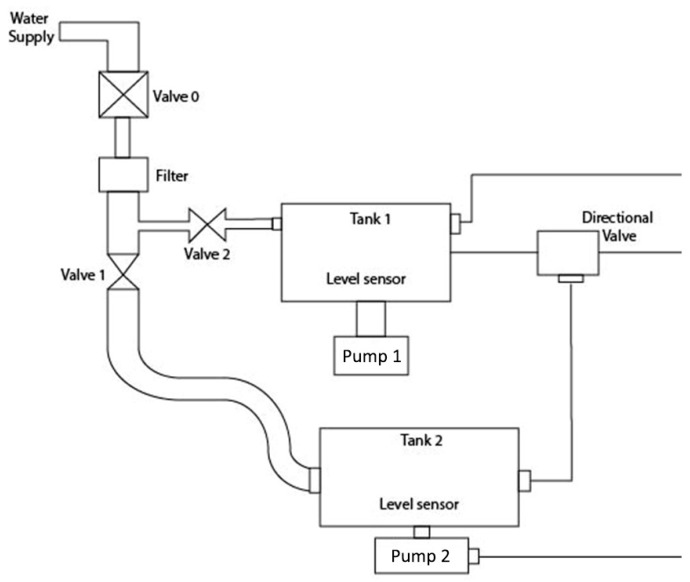
Global scheme of the system.

**Figure 12:**
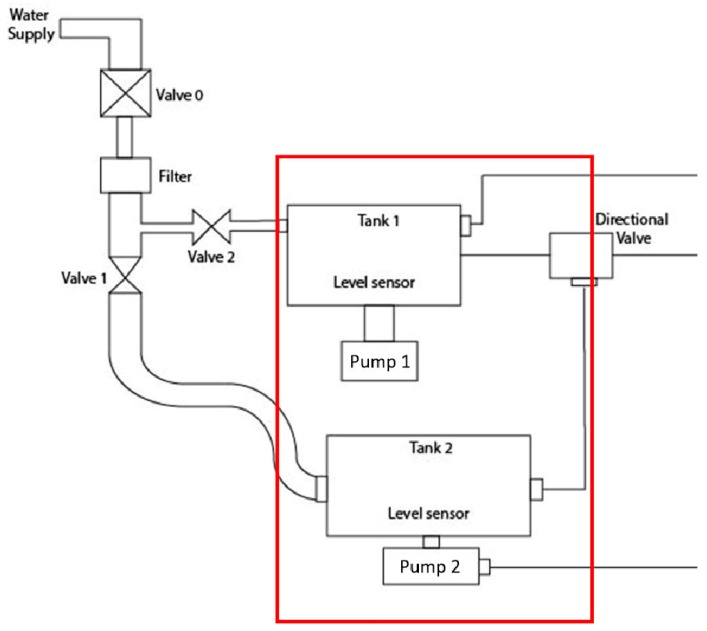
Level sensor scheme.

The total scheme of the system is a model which shows the internal operation of the device, as can be identified in Figure 11 using two important sensors: a temperature sensor and a level sensor, which aim to ensure the safety of the integrated system. The sensors used in this system can be exemplified to discriminate the respective characteristics that were applied in this device. Figure 12, shows the scheme where two level sensors are used in the two different water supply tanks.

**Figure 13:**
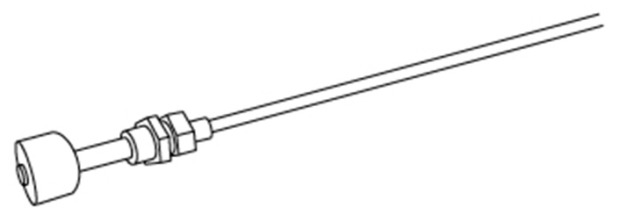
Float liquid level sensor (adapted from [39]).

The level sensor has been implemented to ensure the amount of water and the water tank filler limit. The Float Liquid Level Sensor chosen was the STANDEXMEDER [36] (Figure 13). The material of construction is nylon and some of the characteristics indicates that may be used in temperature up 80 °C, the tightening torque was 0.5 N/m and the floating specific density approximately 0.7 g/cm^3^. The sensor resistance measured with 40% overdrive was max 280 mOhm.

**Figure 14:**
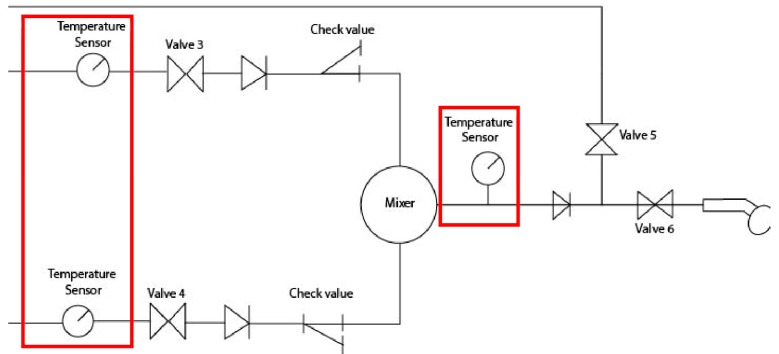
Temperature sensor scheme.

The heating system of this water requires the use of a temperature sensor, Figure 14, to control the temperature variation of the water within the device that must take into account the heating limit of the water that reaches 60 °C internally. This sensor can provide information on tank status, as well as faucet water temperature.

**Figure 15:**
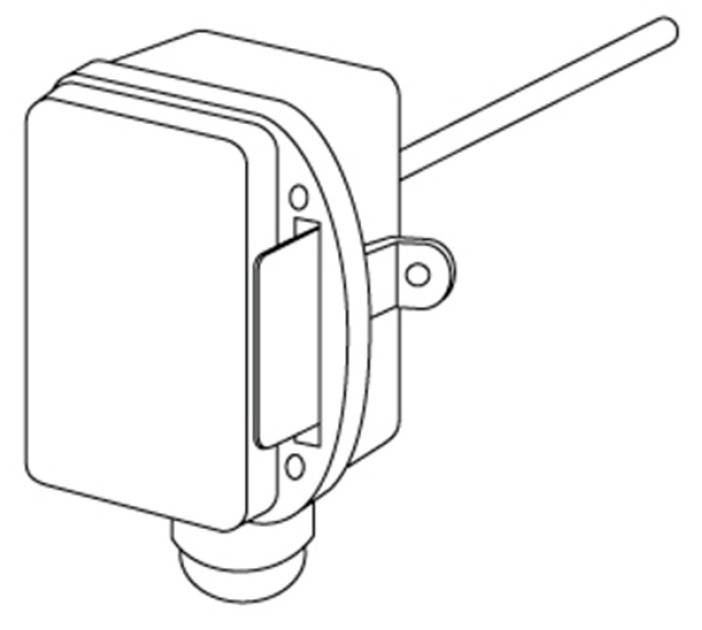
Soaking temperature probe (PTT-341) (adapted from [40]).

In order to guarantee water heating safety, three temperature sensors are used throughout the system. Soaking temperature probes (PTT-341 [40]) were implemented (Figure 15), to measure the temperature of the liquid internals still in the pipeline. They use a high quality thermistor and a nickel sensor element, and have the capacity to measure high temperatures above 90 °C.

## 4. Validation of the Concept

It should be considered that this mechatronic system is still in development. However, the validation section of this device requires a methodological sequence of tasks for designing prototype validation on a 1:1 scale. The tests will be performed during the bath, through the caregivers who are responsible for the procedure. One can describe the steps that will be followed for device usage validation. The following will be analyzed:

- Device installation time in the care environment;
- The portability of the equipment;
- Filling time;
- Testing the water temperature in the external environment;
- Process control (time, temperature, pressure, configuration conditions, etc.);
- Software Parameters;
- Process Operation Procedures;
- Potential failure modes;
- Product Acceptability;
- Process Stability.

The use of the devices in the intended use environment must be taken into account. The environment, chosen by the caregiver, might be the bedroom or the bathroom. The following validation steps should be considered:

1st step: Supply the water tank;
2nd step: Move to the care environment;
3rd step: Test to Turn on the device;
4th step: Test Control Interface;
5th step: Adjust device for use
6th step: Start counting the usage time;
7th step: Output water temperature test;
8th step: Length test and shower use;
9th step: End the bath;
10th step: Stop time counting.

The test aims to validate formal and functional aspects such as: ergonomics, interface, usability and time of use.

## 5. Results

The system is supported by a structure with three multidirectional wheels that allows the movement of the device.

**Figure 16:**
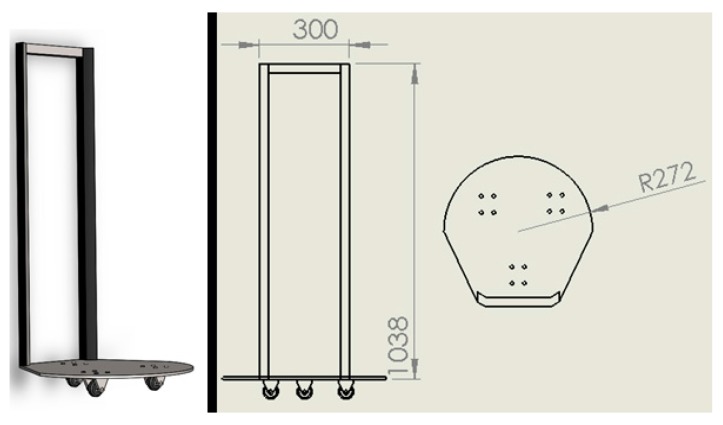
Support structure (unit: mm).

The structure was developed with the aim of providing stability during the movement of the device, considering impacts. Figure 16 presents the designed structure.

**Figure 17:**
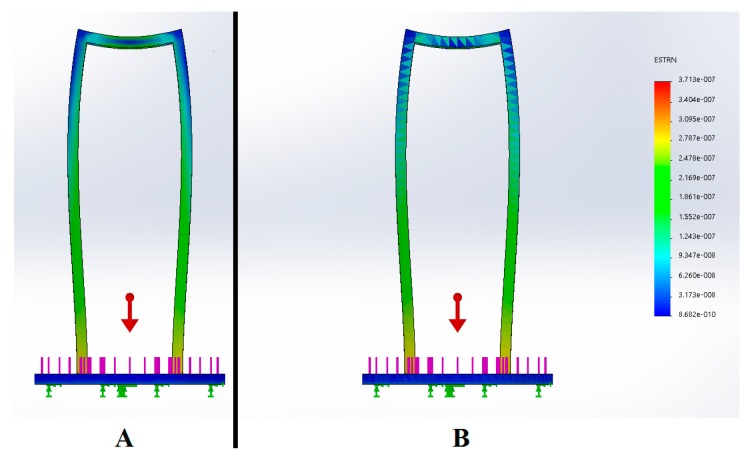
Simulation study: (**A**) Stress, (**B**) Strain.

The structure was made of stainless steel and designed to support a maximum mass of 240 kg (2400 N). The typical safety factor of medical devices—three times the system normal mass—was considered. Using the Solidworks software some simulations were performed with the aim of observing the structure behavior when a load is applied (Figure 17).

### 5.1. Shower

Physiologically, bathing allows cleaning and scrubbing dead skin, preventing irritations and rashes that could become infections. It allows people to remain clean and relaxed through the whole washing process. For this, and because the intention was to design a product that is not operated by the user, but by a caretaker, a showerhead that was easy to use and offered a quick response was considered [41]. Moreover, the skin of bedridden people is more susceptible to injuries than the skin of active individuals. Thus, a flow and a pressure controller independent from the temperature loop are needed. Based on these requirements, the selected shower was a HotelSpa^®^ AquaCare Series Ultra-Luxury 7—featuring a good quality/price ratio [42].

### 5.2. Interface

**Figure 18:**
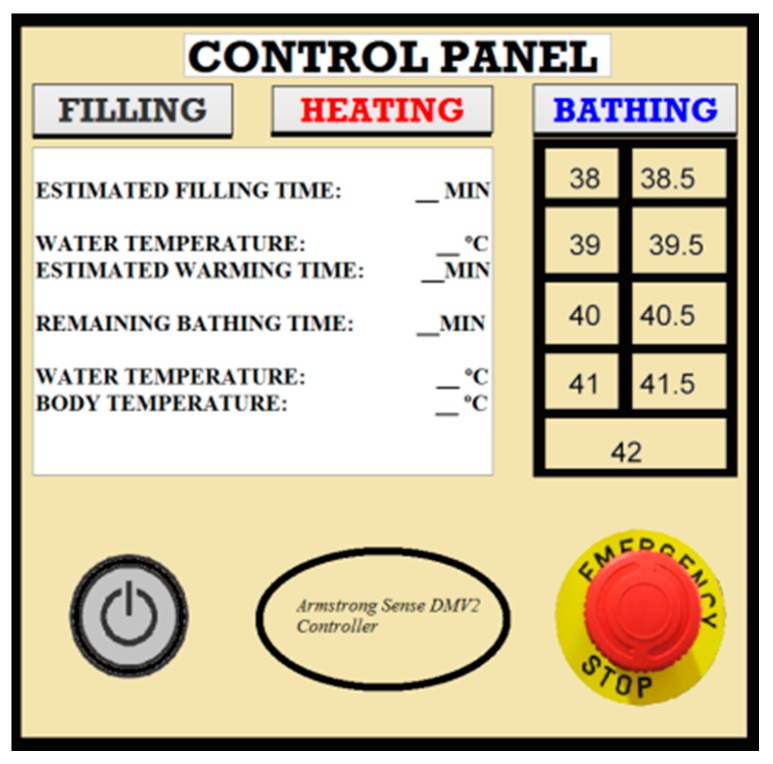
Interface—control panel of the system.

The interface was designed to help the caregiver and provide important information about the bath parameters. It is divided into three parts, one for each main step (Filling, Heating, and Bathing), plus a start button and an emergency button. A numeric panel is used to select the desired temperature and display information about filling, heating and bathing temperature and time. Figure 18 presents the initial design of the control panel. It is composed by the following parts:

Armstrong Sense DMV2 control panel for regulating temperature, presented in Section 3.7.4.

A button for presetting water levels: (a) when the hot water tank is full, the valve between the two tanks closes; (b) starting filling the cold water tank; (c) when the cold water tank is almost full, a light on the control panel turns on, accompanied by a sound warning the caregiver that the tank is almost full; (d) when the tank is full, a sensor gives a signal to interrupt the water flow.

A button for starting the heating of water: (a) when the water temperature reaches 43 °C, a light on the control panel turns on, accompanied by a sound signal warning the caregiver that he or she can start bathing the patient. When the thermostat provides a temperature reading of 43 °C, the heating is stopped;

A button used to initialize/stop the bath;
A button for turning the system on and off;
An emergency button.

### 5.3. Step-by-Step

**Figure 19:**
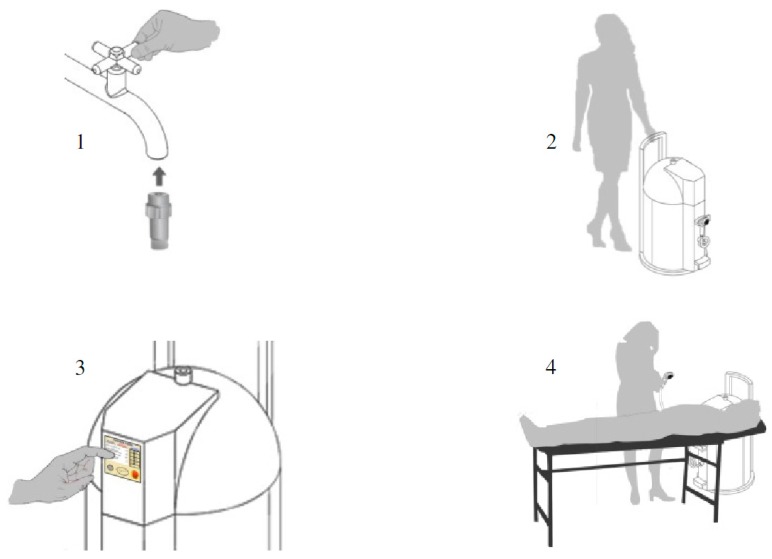
Simulation of use—four total steps.

This section presents the steps that must be followed for an efficient use of the PWS (Figure 19). First, the device must be connected to an external water supply and to be filled until its full capacity. This connection is made with the help of an adaptor between the water outlet of the external water supply and the water inlet of the device (Figure 19-1). The water supply is stopped after accumulating 30 L in the hot water tank, and 15 L in the cold water tank; the user removes the connection between the device and the water supply.

The user pushes the system to the environment where the healthcare task needs to be performed, namely the bed (Figure 19-2).

After connecting the device to the energy supply, the caregiver hits the start button on the control panel to initialize the device (Figure 19-3). He/she must select the parameters of the bath, including the heating mode (1500 W or 3000 W) and the desired water temperature which defines the water flows between the two tanks. While the water is being heated, the caregiver can prepare the bath for the bedridden patient.

After that, the bath system is ready for use. The last step (Figure 19-4) requires that the caregiver perform the activity of bathing the patient. The caregiver can control the shower water flow and pressure.

### 5.4. Concept Design

**Figure 20:**
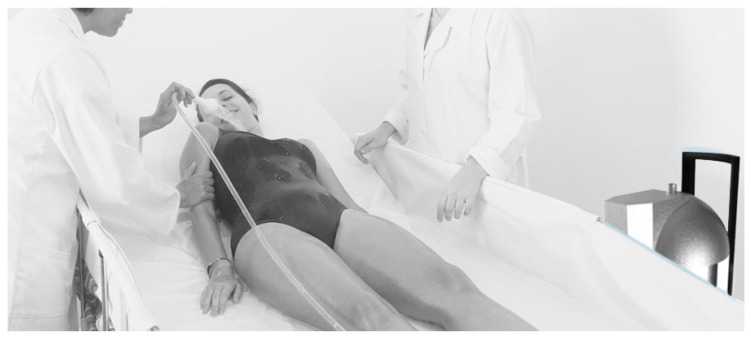
Simulation of use—design solution.

The most important part of the device is its use and the viability of the social concept that is integrated with the functional concept of the designed mechatronic system. In order to improve the quality of life of caregivers and bed rest, the system comprises the various functions and objectives translated into the versatility of this prototype. The simulation of use, illustrated in Figure 20, defines the possibility of a comfortable bath through the use of the bathing system that allows its use by only one person.

## 6. Conclusions

The demographic increase of the elder population and associated social problems are opening a new area in research, in particular for new medical devices designed to fulfill the needs that exist in the medical care for bedridden people. By analyzing the difficulties that exist in taking care of people with disabilities, such as physical effort, dedication and need for equipment, new products need to be developed to help people in attending bedridden patients in a safe, mobile, comfortable, and hygienic environment.

The goal of this project is to develop a Portable Washing System—in short a PSW—to assist in bathing bedridden patients. An important aspect is that the main purpose of the device is to help the caregiver, as the primary user of the device. It is worth mentioning that sometimes only one caregiver is available, often the spouse of the bedridden person.

The presented Portable Washing System concept allows incorporating all the components necessary for the operation of the system according to the defined specifications to help caregivers in daily tasks. The aim of this project is to provide a benefit in terms of technical device innovation and to facilitate bathing patients in bed minimizing the repetitive processes caregivers must perform in daily care. Moreover, when compared to current commercial systems, is an interesting solution for underdeveloped countries where a fragile economy may not allow the acquisition of more sophisticated and costly devices.

In the future, we will consider building a physical prototype (taking into account caregivers’ opinions) and testing it in a controlled environment. The final step is to test it in institutions and private homes for improving system performance.

## Figures and Tables

**Table 1:** Requirements and respective specifications of product development.

Requirements	Specifications
Water Temperature	The selected controller allows choosing temperatures between 38 °C and 43 °C
Thermal Insulation	Polyurethane insulation—loss ratio of 1 °C in 3 h
Ecological	Conventional showers have a flow rate of 7.5 L/min, while this system has an operating rate of 5 L/min
Water Heating	Heating time: 22 min
Hygiene	Customized with a water filter for impurities in the water. The modular structure enables the user to have an easily access to the filter to permit a regular cleaning of the filter
Portability	Support structure, with a base constituted by three multi-directional wheels

**Table 2:** Morphological Chart [30].

Functions		Solutions		
**1**	Wheel	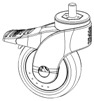	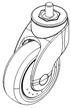	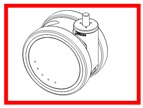
**2**	Capacity of water	10 L	20 L	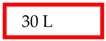
**3**	Support	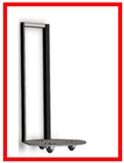	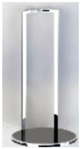	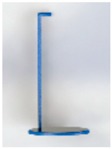
**4**	Mixing Valve	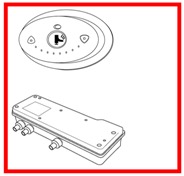	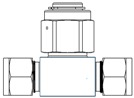	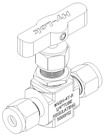
**5**	Water Filter	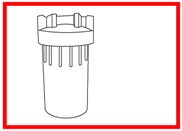	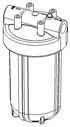	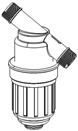
**6**	Shower Head	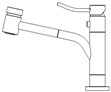	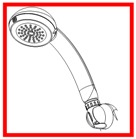	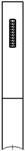

**Table 3:** Specifications of the heated tank.

Temperature (°C)		Dimensions (mm)		Capacity (L)
Water	Environment	Diameter	Height	
42	10	360	400	8.5

**Table 4:** Properties of the Materials Used, Water and Air Coefficients.

Water Specific Heat (Cp)	4872 J/kg·K
Water convection coefficient (hw)	8.0567 W/m^2^·K
Air convection coefficient (hair)	3640 W/m^2^·K
Stainless steel thermal conductivity (kss)	15 W/m·K
Polyurethane thermal conductivity (kpu)	0.035 W/m·K
